# ﻿Taxonomic review of *Amemboa* Esaki, 1925 from China, with description of a new species (Hemiptera, Heteroptera, Gerridae)

**DOI:** 10.3897/zookeys.1210.125611

**Published:** 2024-08-15

**Authors:** Zhaoqi Leng, Beichen Zhang, Zezhong Jin, Zhen Ye

**Affiliations:** 1 Institute of Entomology, College of Life Sciences, Nankai University, Tianjin, 300071, China Nankai University Tianjin China; 2 Laboratory of Entomology, Wageningen University and Research, Wageningen, 6700 AA, Netherlands Wageningen University and Research Wageningen Netherlands

**Keywords:** *Amemboa*, Eotrechinae, Hainan, new records, taxonomy

## Abstract

The species of *Amemboa* Esaki, 1925 from China are reviewed. *Amemboahainanica***sp. nov.**, is described from Hainan Island, and *Amemboaburmensis* J. Polhemus & Andersen, 1984 is newly recorded from China. Additionally, diagnoses and new distribution records are provided for six species previously reported from China: *A.brevifasciata* Miyamoto, 1967, *A.cristata* J. Polhemus & Andersen, 1984, *A.esakii* J. Polhemus & Andersen, 1984, *A.fumi* Esaki, 1925, *A.riparia* J. Polhemus & Andersen, 1984, and *A.speciosa* J. Polhemus & Andersen, 1984. Photographs and line drawings of the habitus, the diagnostic characteristics of males, in-situ habitus, and their habitats are presented. A key and a distribution map are also provided for species of *Amemboa* occurring in China.

## ﻿Introduction

*Amemboa* Esaki, 1925 belongs to the water strider subfamily Eotrechinae. Members of this genus typically inhabit the edges of slow-flowing rivers or still ponds near rivers. The species of *Amemboa* are found in South and Southeast Asia, ranging from India to the Philippines, and exhibit a notable diversity in tropical regions (Polhemus and Andersen 1984; Zettel 1995, 1998, 2002; Zettel and Chen 1996, 1997; Zettel et al. 2007; Polhemus and Tran 2012; Basu et al. 2014; Polhemus 2017; Jehamalar et al. 2023). Jehamalar et al. (2023) classified *Amemboa* into 11 species groups based on the morphological characteristics of males. Prior to this study, *Amemboa* comprised a total of 28 described species (Jehamalar et al. 2023).

Six *Amemboa* species were recorded from China: Polhemus and Andersen (1984) described two species from Taiwan Island, namely *A.esakii* J. Polhemus & Andersen, 1984 and *A.fumi* Esaki, 1925. Cheng et al. (2006) reported the distribution of “*A.lyra* (Paiva, 1918)” in Yunnan. However, Zettel et al. (2007) re-examined the specimens of Cheng et al. (2006) and found that the profemora of males correspond to *A.riparia* J. Polhemus & Andersen, 1984. *Amemboariparia* was recognized as a synonym of *A.lyra* by Zettel and Chen (1996) and subsequently re-established as a valid species by Polhemus and Tran (2012). Therefore, the species reported by Cheng et al. (2006) was probably *A.riparia*, while *A.lyra* had no reliable distribution records from China. Additionally, Zettel et al. (2007) first reported the distribution of *A.brevifasciata* Miyamoto, 1967 and *A.speciosa* J. Polhemus & Andersen, 1984 in Hainan, and *A.cristata* Polhemus & Andersen, 1984 in Yunnan.

This study describes a new species, *Amemboahainanica* sp. nov., from Hainan, China. The study also reports the distribution of *A.burmensis* J. Polhemus & Andersen, 1984 in China for the first time. New distribution data are provided for seven *Amemboa* species. Diagnoses, photographs of diagnostic characteristics, a distribution map, and a key are provided for all *Amemboa* species distributed in China. Line drawings, in-situ habitus photographs, and photographs of habitats are also included.

## ﻿Materials and methods

All measurements are given in millimeters (mm), representing the average values of the measurements taken from the type specimens. Measurements, observations, and dissections were made using a Zeiss Discovery V8 stereo microscope. Male genitalia were macerated in 5% potassium hydroxide solution (KOH) at room temperature. Photographs of male genitalic structures (pygophore and proctiger) were taken by using a Canon 90D camera equipped with a micro lens. All other photographs except for the male genitalic structures were taken with a Nikon D500 camera equipped with a micro lens and a telephoto lens. The map was prepared using ArcMap v. 10.8 software.

Dried and alcohol-preserved specimens examined in this study have been deposited in Institute of Entomology, College of Life Sciences, Nankai University, Tianjin, China (**NKUM**).

## ﻿Taxonomic accounts

**Family Gerridae Leach, 1815**

**Subfamily Eotrechinae Matsuda, 1960**

**Genus *Amemboa* Esaki, 1925**

### ﻿ Amemboa hainanica

Taxon classificationAnimaliaHemipteraGerridae

Leng, Jin & Ye sp. nov.

6F0F49B5-40CD-50F4-B2DC-81033D3F9ABB

https://zoobank.org/7D2BD0D7-CDAA-4523-90FB-0C54783AF425

Figs 1 , 2A, B , 3A , 4A, B, E, F , 5 , 6A , 7A , 8A , 9A , 10A , 11A , 12A, B , 15A , 16 , 18

#### Material examined.

***Holotype***: ♂ (apterous), China, Hainan Province, Bai-sha County, Luo-shuai Village, Xian-nv-xi; 19°5'58.4"N, 109°32'46.2"E; 324 m a.s.l.; 26 Jul. 2017; Zhen Ye & Juan-juan Yuan leg. (NKUM). ***Paratypes***: 3 ♂♂, 3 ♀♀ (apterous), same data as holotype (NKUM) • 3 ♂♂, 3 ♀♀ (apterous), China, Hainan Province, Wan-ning City, Xing-long County, Ai-qing-gu; 18°47'54.3"N, 110°8'19.8"E; 148 m a.s.l.; 11 Aug. 2017; Juan-juan Yuan leg. (NKUM) • 1 ♂, 1 ♀ (apterous), China, Hainan Province, Bao-ting County, Qi-xian-ling National Forest Park; 18°42'12.2"N, 109°41'44.5"E; 318 m a.s.l.; 4 Aug. 2017; Zhen Ye leg. (NKUM) • 1 ♂, 1 ♀ (apterous), China, Hainan Province, Le-dong County, Jian-feng-ling, Wu-fen-he, Pool 3; 18°43'58.0"N, 108°53'9.6"E; 859 m a.s.l.; 15 Aug. 2017; Juan-juan Yuan leg. (NKUM) • 1 ♂, 1 ♀ (apterous), China, Hainan Province, Qiong-zhong County, Li-mu-shan; 19°11'11.1"N, 109°44'21.4"E; 631 m a.s.l.; 25 Jul. 2017; Zhen Ye leg. (NKUM) • 1 ♂, 1 ♀ (apterous), China, Hainan Province, Bai-sha County, Luo-shuai Village, Xian-nv-xi; 19°5'58.4"N, 109°32′46.2"E; 324 m a.s.l.; 26 Jul. 2017; Zhen Ye & Juan-juan Yuan leg. (NKUM) • 1 ♂, 1 ♀ (apterous), China, Hainan Province, Wan-ning City, Jian-feng-ling Nature Reserve, Yu-lin-gu; 18°42'30.9"N, 108°49'20.2"E; 1 Aug. 2017; Zhen Ye leg. (NKUM) • 1 ♂, 1 ♀ (apterous), China, Hainan Province, Qiong-zhong County, Beng-ling Village; 18°46'42.7"N, 109°50'29.3"E; 253 m a.s.l.; 9 Aug. 2017; Zhen Ye leg. (NKUM) • 1 ♂, 1 ♀ (apterous), China, Hainan Province, Wan-ning City, Xing-long County, Ai-qing-gu; 18°47'54.3"N, 110°8'19.8"E; 148 m a.s.l.; 11 Aug. 2017; Juan-juan Yuan leg. (NKUM).

***Non-type materials***: 1 ♂ (apterous), China, Hainan Province, Chang-jiang County, Ba-wang-ling Nature Reserve; 19°7'16.8"N, 109°9'38.2"E; 650 m a.s.l.; 6 Apr. 2008; Bo Cai leg. (NKUM) • 1 ♂ (apterous), China, Hainan Province, Bao-ting County, Xian-an-shi-lin; 18°36'5.8"N, 109°25'59.5"E; 571 m a.s.l.; 7 Aug. 2017; Kun Jiang & Si-ying Fu leg. (NKUM) • 2 ♂♂, 1 ♀ (apterous), China, Hainan Province, Bai-sha County, Shi-cai Village, Ying-ge-ling Nature Reserve, Nan-kai Branch Station; 19°4'23.0"N, 109°22'34.0"E; 310 m a.s.l.; 20 Jul. 2013; Yan-hui Wang leg. (NKUM) • 3 ♂♂ (apterous), China, Hainan Province, Dan-zhou City, Lu-mu-wan Waterfall; 19°13'54.6"N, 109°41'16.5"E; 202 m a.s.l.; 22 Jul. 2017; Zhen Ye leg. (NKUM) • 3 ♂♂, 8 ♀♀ (apterous), China, Hainan Province, Tun-chang County, Nan-lv-ling; 19°13'49.0"N, 110°10'20.8"E; 172 m a.s.l.; 24 Jul. 2017; Zhen Ye leg. (NKUM) • 1 ♂ (apterous), China, Hainan Province, Wu-zhi-shan City, Shui-man Township, Shi-ling-rui Bridge; 18°54'10.9"N, 109°39'23.7"E; 3 Jul. 2014; Qiang Xie leg. (NKUM) • 3 ♂♂, 6 ♀♀ (apterous), China, Hainan Province, Bai-sha County, Ying-ge-ling Nature Reserve, Nan-kai Branch Station; 19°2'1.7"N, 109°24'14.5"E; 20 Jul. 2013; Hao-yang Wu leg. (NKUM).

#### Diagnosis.

**Figure 1.**
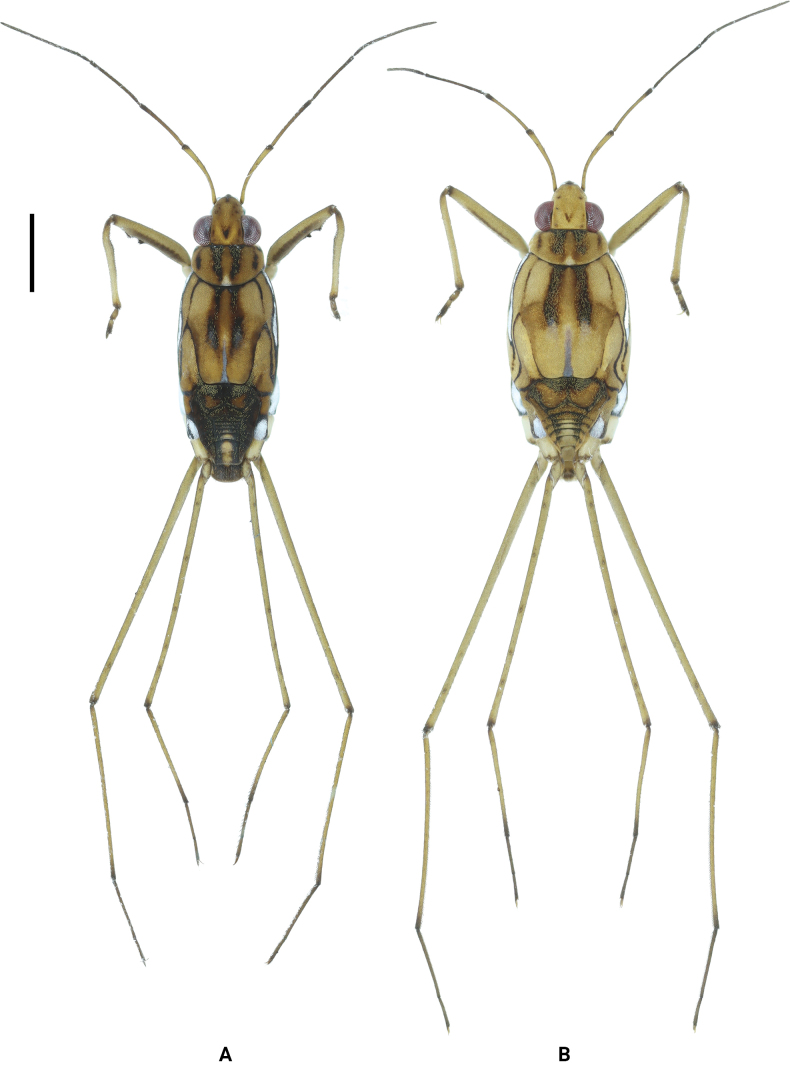
Habitus of *Amemboahainanica* sp. nov., apterous form in dorsal view **A** holotype, male **B** paratype, female. Scale bar: 2 mm.

**Figure 2.**
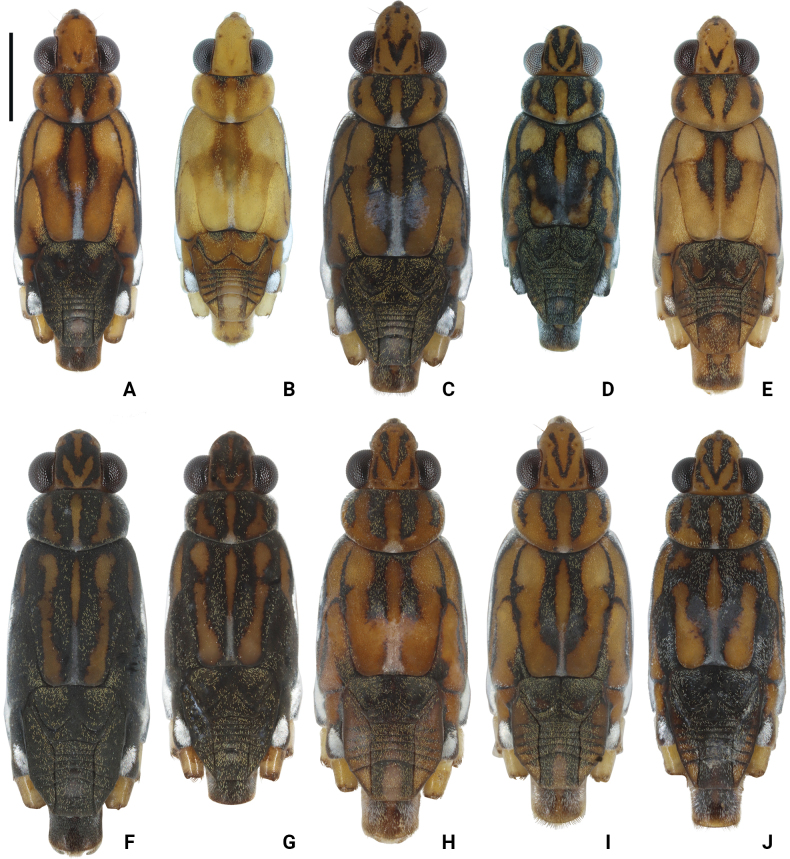
Photographs of bodies of *Amemboa* spp., apterous males in dorsal view **A, B***A.hainanica* sp. nov **C***A.brevifasciata* Miyamoto, 1967 **D***A.burmensis* J. Polhemus & Andersen, 1984 **E***A.cristata* Polhemus & Andersen, 1984 **F***A.esakii* J. Polhemus & Andersen, 1984 **G***A.fumi* Esaki, 1925 **H, I***A.riparia* J. Polhemus & Andersen, 1984 **J***A.speciosa* J. Polhemus & Andersen, 1984. Scale bar: 1 mm.

**Figure 3.**
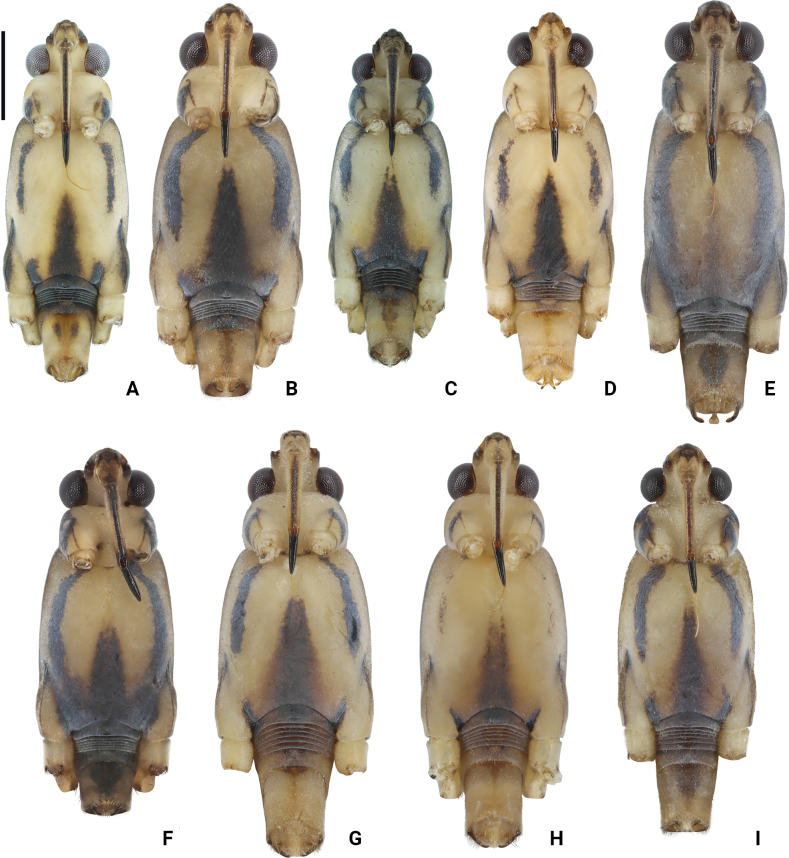
Photographs of bodies of *Amemboa* spp., apterous males in ventral view **A***A.hainanica* sp. nov. **B***A.brevifasciata* Miyamoto, 1967 **C***A.burmensis* J. Polhemus & Andersen, 1984 **D***A.cristata* Polhemus & Andersen, 1984 **E***A.esakii* J. Polhemus & Andersen, 1984 **F***A.fumi* Esaki, 1925 **G, H***A.riparia* J. Polhemus & Andersen, 1984 **I***A.speciosa* J. Polhemus & Andersen, 1984. Scale bar: 1 mm.

**Figure 4.**
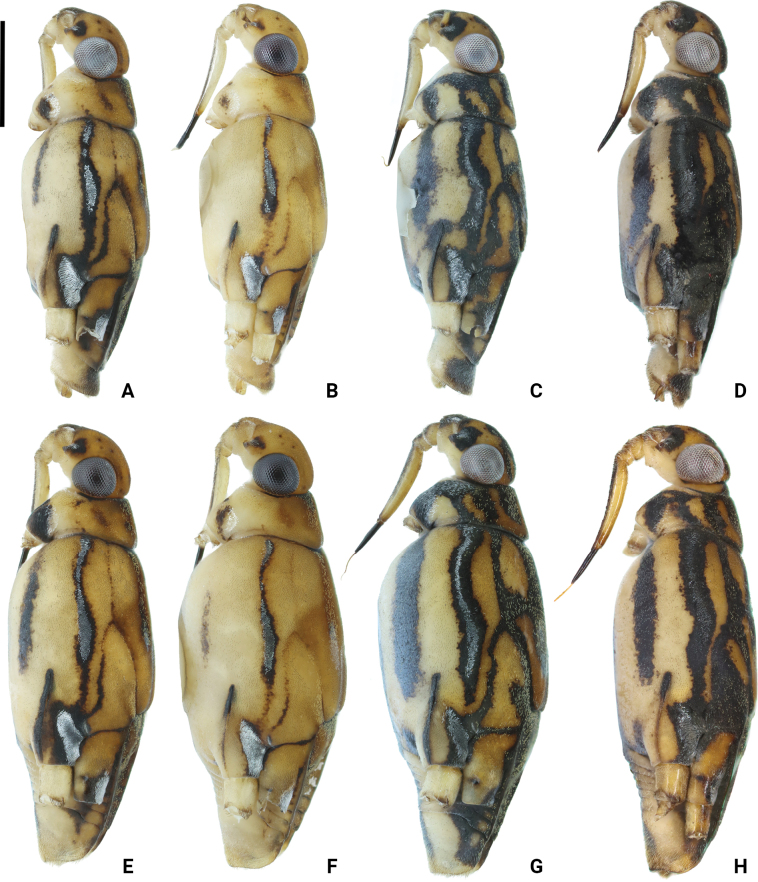
Photographs of bodies of *Amemboa* spp., apterous form in lateral view **A, B***A.hainanica* sp. nov., males **C***A.burmensis* J. Polhemus & Andersen, 1984, male **D***A.fumi* Esaki, 1925, male **E, F***A.hainanica* sp. nov., females **G***A.burmensis*, female **H***A.fumi*, female. Scale bar: 1 mm.

**Figure 5.**
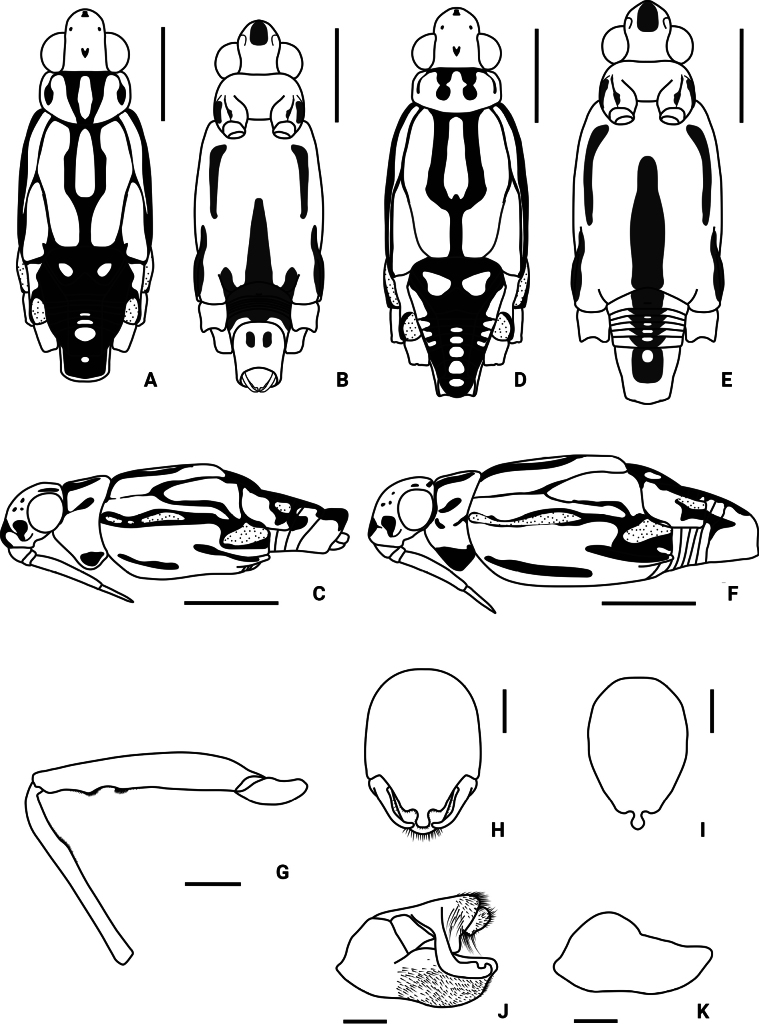
Morphological features of *Amemboahainanica* sp. nov. **A** body of male, dorsal view **B** body of male, ventral view **C** body of male, lateral view **D** body of female, dorsal view **E** body of female, ventral view **F** body of female, lateral view **G** left foreleg of male, dorsal view **H** pygophore and proctiger of male, ventral view **I** pygophore of male, ventral view **J** pygophore and proctiger of male, lateral view **K** pygophore of male, lateral view. Scale bars: 1 mm (**A–F**); 0.2 mm (**G**); 0.1 mm (**H–K**).

**Figure 6.**
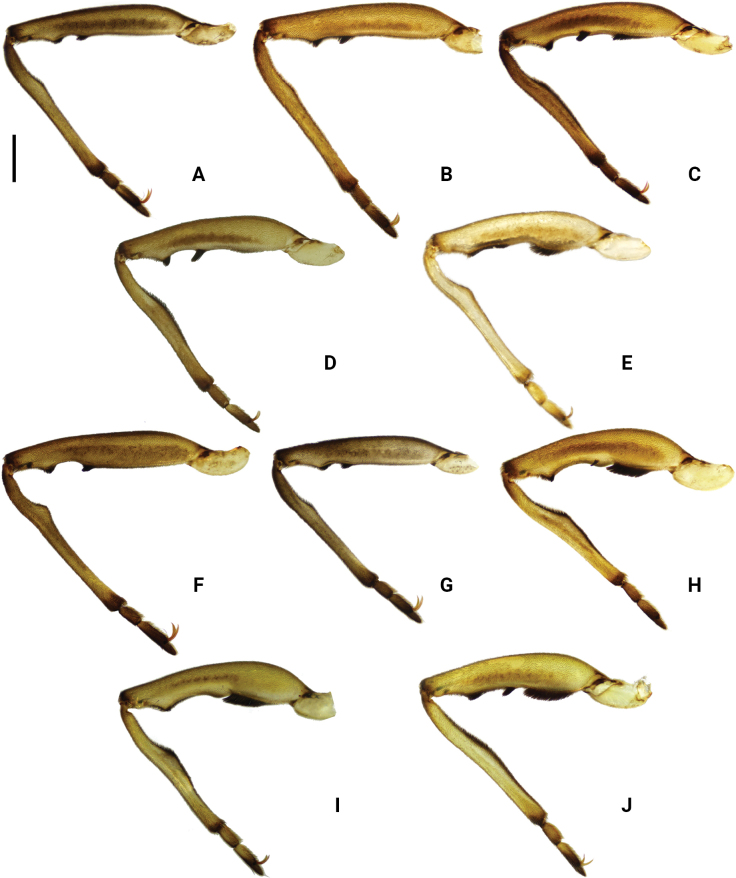
Left forelegs of *Amemboa* spp. in dorsal view (males) **A***A.hainanica* sp. nov. **B***A.brevifasciata* Miyamoto, 1967 **C, D***A.burmensis* J. Polhemus & Andersen, 1984 **E***A.cristata* Polhemus & Andersen, 1984 **F***A.esakii* J. Polhemus & Andersen, 1984 **G***A.fumi* Esaki, 1925 **H, I***A.riparia* J. Polhemus & Andersen, 1984 **J***A.speciosa* J. Polhemus & Andersen, 1984. Scale bar: 0.2 mm.

Color pattern as shown in Figs 1, 2A, B, 3A, 4A, B, E, F, 5A–F, 12A, B. Males: profemur moderately incrassate (Figs 5G, 6A); ventral side of the profemur with two tufts of dark setae on apical 1/2 (Figs 5G, 6A); protibia slightly curved and with an indistinct tumescence on basal 1/3 (Figs 5G, 6A); abdominal segment VIII relatively short (Fig. 8A); pygophore posteriorly with a digitate median process, also with a pair of distinct blunt processes on both sides of the median process (Figs 5H, I, 8A, 9A); median process of pygophore relatively broad in lateral view (Figs 5J, K, 10A, 11A); lateral arm of proctiger relatively slender in ventral view (Figs 5H, 8A), with a distinct subapical process in lateral view, forming a distinct subapical notch on dorsal margin (Figs 5J, 10A).

#### Comparative notes.

*Amemboahainanica* sp. nov. is most similar to *A.cambodiana* D. Polhemus, 2017. In males of both species, the lateral arm of the proctiger in lateral view exhibits a distinct subapical notch on its dorsal margin, isolating a small, slightly recurved apical process, which gives the lateral arm a hooked appearance (Fig. 10A; also see Polhemus 2017: fig. 22). These are the only two known *Amemboa* species that share this morphological characteristic. Besides, the ventral side of the profemur bears two tufts of dark setae in the males of both species (Fig. 6A; also see Polhemus 2017: fig. 19). Some color patterns of both species are also similar (Figs 2A, 12A; also see Polhemus 2017: figs 14, 15). However, these two species can be distinguished by the following characteristics. The apical hook of lateral arm is more acutely pointed in *A.cambodiana* (see Polhemus 2017: fig. 22), while it is blunter in *A.hainanica* sp. nov. (Fig. 10A). The subapical process is weakly developed in *A.cambodiana*, while it is more distinct in *A.hainanica* sp. nov. (Fig. 10A; also see Polhemus 2017: fig. 22). The two species can also be separated by the shape of their pygophores, which is campanulate in *A.cambodiana* (see Polhemus 2017: fig. 21) and lacks the pair of lateral blunt processes seen in *A.hainanica* sp. nov. (Fig. 9A).

**Figure 7.**
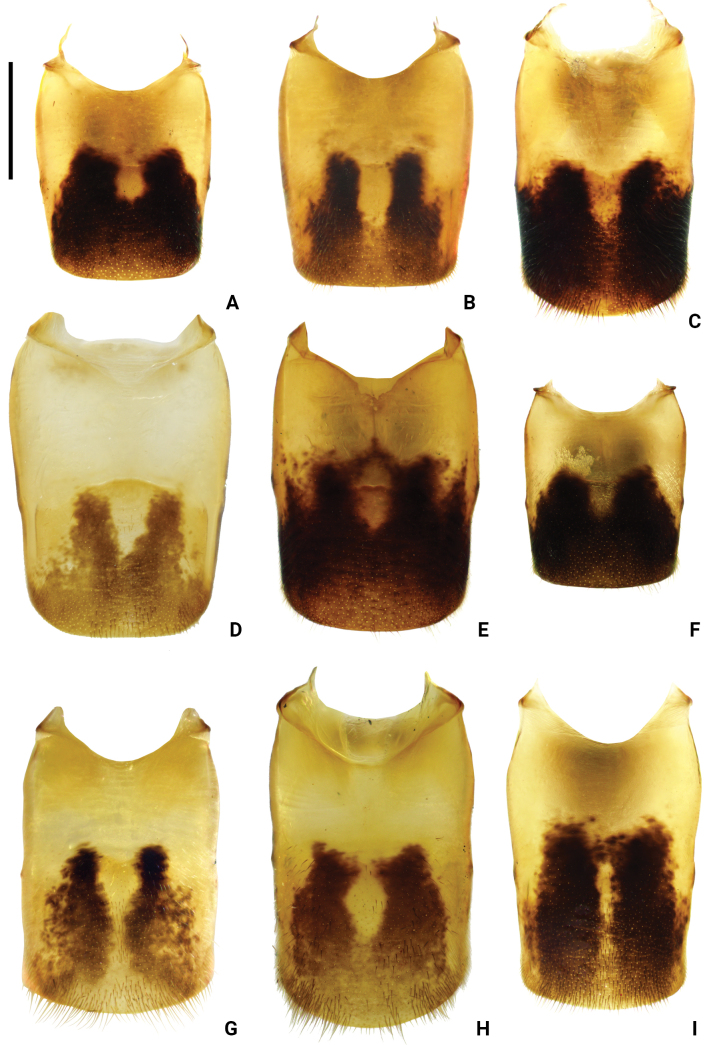
Abdominal segment VIII of *Amemboa* spp. in dorsal view (males) **A***A.hainanica* sp. nov. **B***A.brevifasciata* Miyamoto, 1967 **C***A.burmensis* J. Polhemus & Andersen, 1984 **D***A.cristata* Polhemus & Andersen, 1984 **E***A.esakii* J Polhemus & Andersen, 1984 **F***A.fumi* Esaki, 1925 **G, H***A.riparia* J. Polhemus & Andersen, 1984 **I***A.speciosa* J. Polhemus & Andersen, 1984. Scale bar: 0.2 mm.

*Amemboahainanica* sp. nov. is also similar to *A.fumi* Esaki, 1925. In males of both species, the ventral side of the profemur bears two tufts of dark setae on apical 1/2, and protibia exhibits an indistinct tumescence on the basal 1/3 (Fig. 6A, G); abdominal segment VIII relatively short (Fig. 7A, F); pygophore has a digitate median process posteromedially and a pair of distinct blunt processes to either side of the median process (Fig. 9A, F). However, *A.hainanica* sp. nov. differs from *A.fumi* in the following characteristics. In males of *A.hainanica* sp. nov., the lateral arm of proctiger bears a distinct subapical process in lateral view (Fig. 10A), while in *A.fumi*, the lateral arm of proctiger lacks a process in lateral view (Fig. 10F). Additionally, although color characteristics are quite variable within *Amemboa* species, some generalizations can still be made based on the current specimens. In both sexes of *A.hainanica* sp. nov., the black marks and stripes on head, pronotum and mesonotum are more slender and weaker (Figs 2A, 4A, E, 5A, D, 12A), or nearly absent (Figs 2B, 4B, F, 12B), while they are broader and more prominent in *A.fumi* (Figs 2G, 12G). Moreover, the metanotum of *A.hainanica* sp. nov. exhibits brownish-yellow spots (Figs 2A, 4A, E, 5A, D, 12A) or is predominantly brownish-yellow (Figs 2B, 4B, F, 12B), whereas it is entirely black in *A.fumi* (Figs 2G, 12G).

#### Description of apterous male.

***Measurements*.** Body length 3.68–3.96 (holotype 3.76), width 1.39–1.49 (holotype 1.39), head width 0.92–0.98, interocular width 0.43–0.44, eye length (in dorsal view) 0.39–0.43; lengths of antennal segments I–IV: 0.86–0.93: 0.78–0.82: 0.83–0.89: 1.32–1.51; pronotum: length 0.53–0.55, width 0.99–1.11; mesonotum: length 1.25–1.30, width 1.39–1.49; metanotum: length 0.44–0.48, width 1.11–1.17; lengths of leg segments (femur: tibia: tarsal segment I: tarsal segment II): fore leg: 1.24–1.30: 1.17–1.21: 0.28–0.29: 0.16–0.18, middle leg: 3.47–3.56: 2.19–2.25: 0.81–0.87: 0.40–0.43, hind leg: 3.05–3.28: 1.22–1.33: 0.48–0.53: 0.35–0.38.

***Color*.** Body dorsally brownish yellow with black stripes (Figs 1A, 2A, B, 5A). Color pattern as shown in Figs 2A, B, 3A, 4A, B, 5A–C. Head dorsally with several tiny, discontinuous, black spots (Figs 2A, B, 5A); rostrum mainly yellow, with dorsomedian line and segment IV black (Fig. 4A, B). Pronotum with two pairs of black stripes in dorsal view (Figs 2A, 5A), sometimes the pair of lateral stripes nearly absent (Fig. 2B); proacetabulum with a pair of black or brown marks laterally, coxal cleft brown (Figs 3A, 5B). Mesonotum with broad, black stripes medially and slender, black stripes laterally (Figs 2A, 4A, 5B, C), sometimes stripes nearly absent (Figs 2B, 4B); mesopleuron with a relatively broad, black stripe medially on each side, covered by silvery setae (Figs 4A, B, 5C); mesacetabulum with broad, black mark dorsally, covered by silvery setae (Figs 2A, B, 4A, B), basal part of coxal cleft black, other part brownish-black (Figs 3A, 4A) or pale brown (Fig. 4B); mesosternum with a pair of elongate, black stripes anterolaterally, a broad, black stripe medially, and a pair of black spots posterolaterally (Figs 3A, 5B). Metanotum mainly black, with a pair of dark brown spots (Figs 2A, 5A) or mainly brownish-yellow (Fig. 2B); metapleuron dark brown or yellowish-brown (Fig. 4A, B); metacetabulum with broad, black mark dorsally, covered by silvery setae (Figs 2A, B, 4A, B); metasternum mainly black (Figs 3A, 5E). Abdominal tergites I–V black (Figs 2A, 5A) or brownish-yellow (Fig. 2B); mediotergites IV–VIII with brownish-yellow spots medially (Figs 2A, B, 5A); abdominal sterna II–VI completely black, sternite VII mainly black with a pair of yellowish-brown stripes on posterior margin (Figs 3A, 5B); abdominal segment VIII mainly yellow, dorsal part black (Figs 2A, 5A) or with brown spots (Fig. 2B), ventral part with a pair of black spots medially (Figs 3A, 5B).

**Figure 8.**
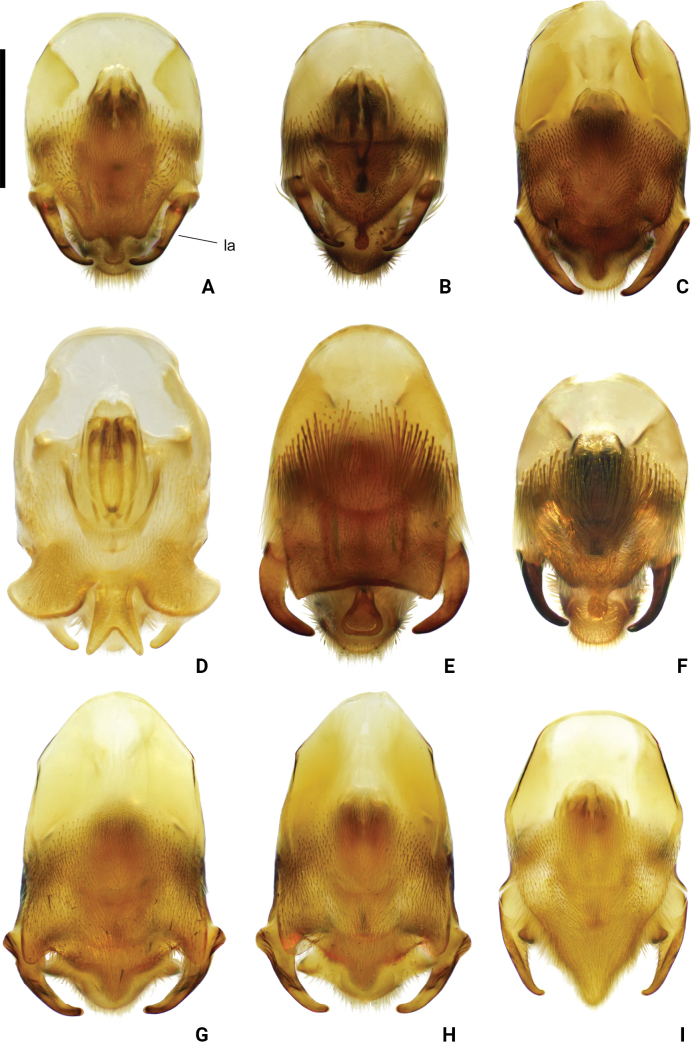
Pygophore and proctiger of *Amemboa* spp. in ventral view **A***A.hainanica* sp. nov. **B***A.brevifasciata* Miyamoto, 1967 **C***A.burmensis* J. Polhemus & Andersen, 1984 **D***A.cristata* Polhemus & Andersen, 1984 **E***A.esakii* J. Polhemus & Andersen, 1984 **F***A.fumi* Esaki, 1925 **G, H***A.riparia* J. Polhemus & Andersen, 1984 **I***A.speciosa* J. Polhemus & Andersen, 1984. Abbreviation: la = lateral arm of proctiger. Scale bar: 0.2 mm.

**Figure 9.**
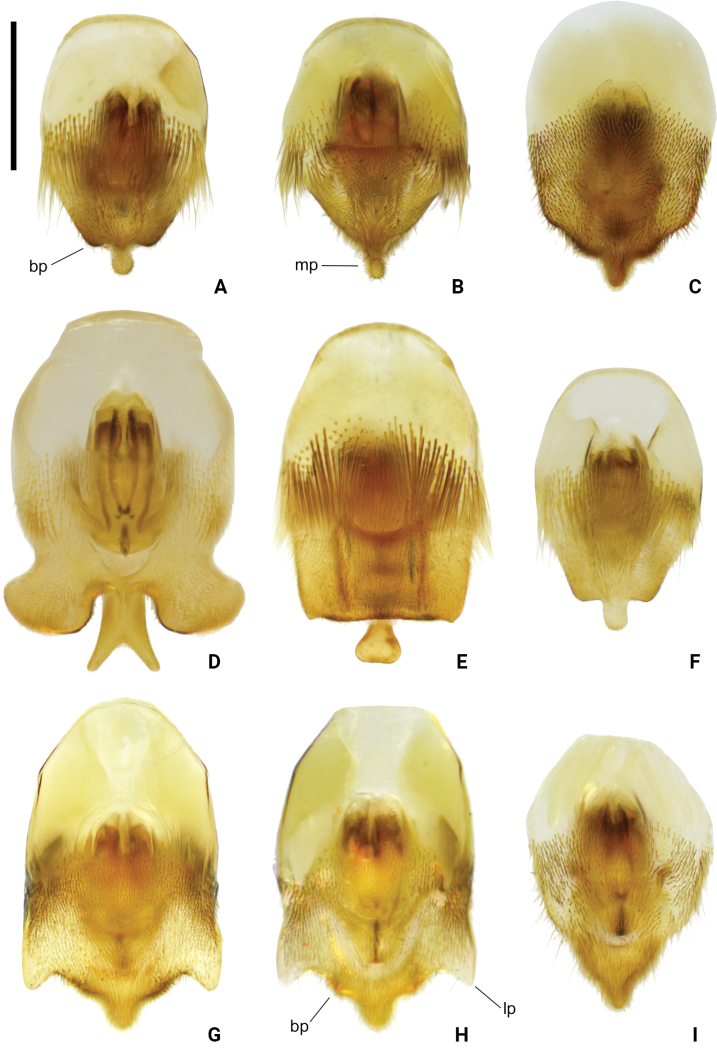
Pygophore of *Amemboa* spp. in ventral view **A***A.hainanica* sp. nov. **B***A.brevifasciata* Miyamoto, 1967 **C***A.burmensis* J. Polhemus & Andersen, 1984 **D***A.cristata* Polhemus & Andersen, 1984 **E***A.esakii* J. Polhemus & Andersen, 1984 **F***A.fumi* Esaki, 1925 **G, H***A.riparia* J. Polhemus & Andersen, 1984 **I***A.speciosa* J. Polhemus & Andersen, 1984. Abbreviations: bp = blunt process, lp = lateral process, mp = median process. Scale bar: 0.2 mm.

**Figure 10.**
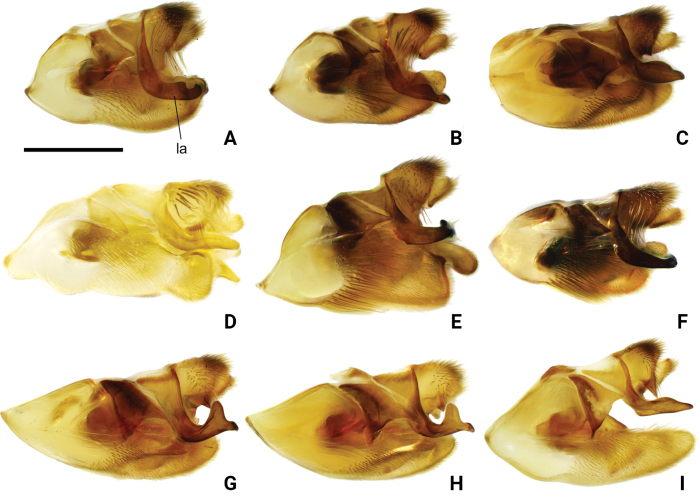
Pygophore and proctiger of *Amemboa* spp. in lateral view **A***A.hainanica* sp. nov. **B***A.brevifasciata* Miyamoto, 1967 **C***A.burmensis* J. Polhemus & Andersen, 1984 **D***A.cristata* Polhemus & Andersen, 1984 **E***A.esakii* J. Polhemus & Andersen, 1984 **F***A.fumi* Esaki, 1925 **G, H***A.riparia* J. Polhemus & Andersen, 1984 **I***A.speciosa* J. Polhemus & Andersen, 1984. Abbreviation: la = lateral arm of proctiger. Scale bar: 0.2 mm.

**Figure 11.**
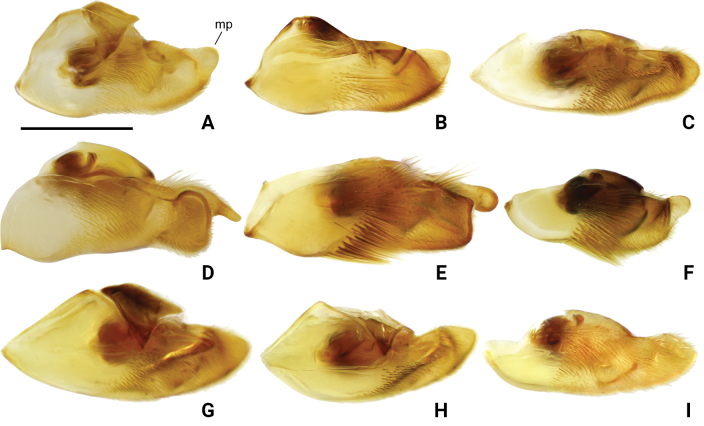
Pygophore of *Amemboa* spp. in lateral view **A***A.hainanica* sp. nov. **B***A.brevifasciata* Miyamoto, 1967 **C***A.burmensis* J. Polhemus & Andersen, 1984 **D***A.cristata* Polhemus & Andersen, 1984 **E***A.esakii* J. Polhemus & Andersen, 1984 **F***A.fumi* Esaki, 1925 **G, H***A.riparia* J. Polhemus & Andersen, 1984 **I***A.speciosa* J. Polhemus & Andersen, 1984. Abbreviation: mp = median process. Scale bar: 0.2 mm.

***Structure*. *Head***: elongate, deflected anteriorly; antennal segment I slightly curved, stouter than other segments, with apical part slightly incrassate, antennal tubercles protruded, visible in dorsal view (Fig. 1A); compound eyes large, globular; rostrum stout, reaching anterior part of mesosternum (Fig. 2A, B). ***Thorax***: prothorax and metathorax short, mesothorax prolonged; proacetabulum oblique, mesoacetabulum and metacetabulum almost horizontal (Figs 4A, B, 5C); profemur moderately incrassate (Figs 5G, 6A); ventral side of the profemur with two tufts of dark setae on apical 1/2 (Figs 5G, 6A); protibia slightly curved, with an indistinct tumescence on basal 1/3 (Figs 5G, 6A); protarsus stout, with large claws (Figs 5G, 6A); meso- and metafemora relatively long, stouter than meso- and metatibia (Fig. 1A); meso- and metatarsi slender (Fig. 1A); claws of middle and hind legs shorter than claw of fore leg (Fig. 1A). ***Abdomen***: mediotergites declined, connexiva raised and convergent (Fig. 2A, B); abdominal segment VII short; abdominal segment VIII cylindriform, protruding from pregenital segments, relatively short and stout (Fig. 7A). ***Genitalia***: pygophore posteriorly with a digitate median process (Figs 5H, I, 8A, 9A), also with a pair of distinct blunt processes on both sides of median process in ventral view (Figs 5H, I, 8A, 9A); median process of pygophore relatively broad in lateral view (Figs 5J, K, 10A, 11A); lateral arms of proctiger relatively slender in ventral view (Figs 5H, 8A), with a distinct subapical process in lateral view, forming a distinct subapical notch on the dorsal margin (Figs 5J, 10A).

#### Description of apterous female.

***Measurements*.** Body length 4.19–4.52, width 1.76–1.78, head width 1.00–1.02, interocular width 0.45–0.49, eye length (dorsal view) 0.44–0.48; lengths of antennal segments I–IV: 0.93–0.99: 0.80–0.88: 0.91–1.00: 1.41–1.54; pronotum: length 0.50–0.55, width 1.01–1.24; mesonotum: length 1.52–1.58, width 1.75–1.78; metanotum: length 0.50–0.56, width 1.32–1.40; lengths of leg segments (femur: tibia: tarsal segment I: tarsal segment II): fore leg: 1.29–1.44: 1.33–1.38: 0.33–0.34: 0.20–0.21, middle leg: 3.88–4.10: 2.50–2.75: 1.02–1.06: 0.46–0.48, hind leg: 3.40–3.53: 1.48–1.56: 0.56–0.64: 0.43–0.46.

**Figure 12.**
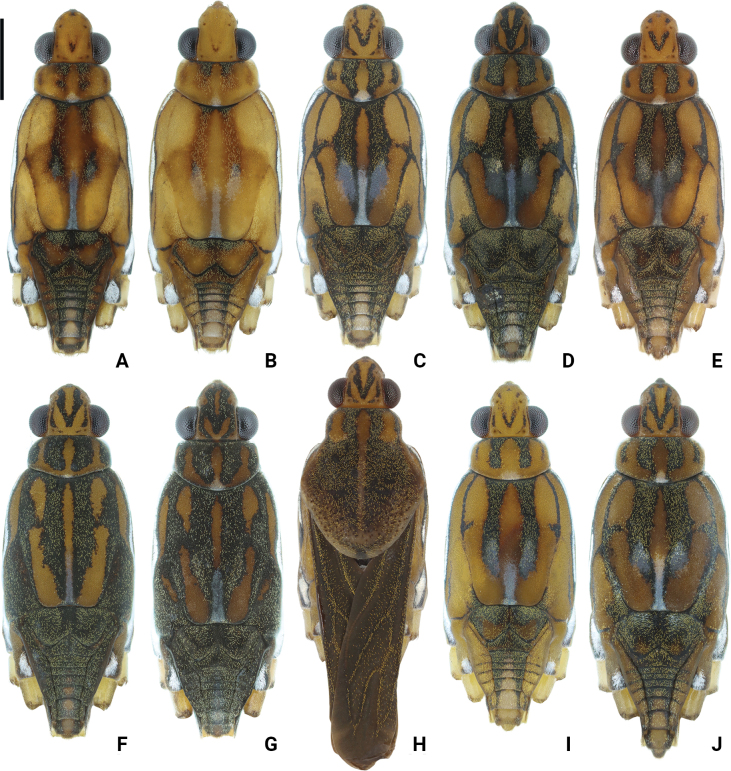
Photographs of bodies of *Amemboa* spp., females in dorsal view, apterous form if not stated otherwise **A, B***A.hainanica* sp. nov. **C***A.brevifasciata* Miyamoto, 1967 **D***A.burmensis* J. Polhemus & Andersen, 1984 **E***A.cristata* Polhemus & Andersen, 1984 **F***A.esakii* J. Polhemus & Andersen, 1984 **G***A.fumi* Esaki, 1925 **H, I***A.riparia* J. Polhemus & Andersen, 1984, macropterous form (**H**) and apterous form (**I**) **J***A.speciosa* J. Polhemus & Andersen, 1984. Scale bar: 1 mm.

***Color*.** Color pattern as shown in Figs 1B, 5D–F, 12A, B. Similar to apterous male with the following exceptions: mesosternum lacking a pair of black spots posterolaterally (Fig. 5E); metasternum mainly yellow with median part black (Fig. 5E); metasternum yellow, with a median black mark (Fig. 5E); abdominal sternites II–VII mainly yellow with median black marks, the marks on sternites III–VII with brown spots in the center (Fig. 5E).

***Structure*.** Similar to apterous male with some exceptions: body stouter than male; profemur slightly incrassate, without special modifications (Fig. 1B); protibia straight without special modifications (Fig. 1B). Abdominal segment VII relatively long, genital segments completely withdrawn into segment VII (Figs 5D–F, 12A, B). Proctiger simple, hind margin rounded.

#### Macropterous forms.

Unknown.

#### Etymology.

The specific epithet *hainanica* refers to the island of Hainan, China, the only area in which this species has so far been collected.

#### Distribution.

China: Hainan (Fig. 18).

#### Habitats.

*Amemboahainanica* sp. nov. is found in rivers and pools below waterfalls (Fig. 15A), actively moving on the water surface (Fig. 16A, B) and resting at the edges of still water puddles (Fig. 16C).

### ﻿ Amemboa brevifasciata

Taxon classificationAnimaliaHemipteraGerridae

Miyamoto, 1967

CB065B7A-B0CA-5116-92D2-FB357057A52F

Figs 2C , 3B , 6B , 7B , 8B , 9B , 10B , 11B , 12C , 18

#### Material examined.

1 ♂, 2 ♀♀ (apterous), China, Guangxi Province, Lai-bin City, Jin-xiu County; 24°8'40.2"N, 110°4'49.1"E; 565 m a.s.l.; 25 Jul. 2019; Zhen Ye leg. (NKUM) • 2 ♂♂ (apterous), China, Guangxi Province, Fang-cheng-gang City, Shang-si County, Shi-wan-da-shan; 21°54'4.0"N, 107°54'22.0"E; 300 m a.s.l.; 13 Jul. 2019; Zhen Ye leg. (NKUM).

#### Diagnosis.

Color pattern as shown in Figs 2C, 3B, 12C. Males: profemur moderately incrassate; ventral side of the profemur with two tufts of dark setae on apical 1/2 (Fig. 6B); protibia slightly curved and with an indistinct tumescence on basal 1/3 (Fig. 6B); abdominal segment VIII relatively short (Fig. 7B); pygophore posteriorly with a digitate median process in ventral view, without other special modifications (Figs 8B, 9B); median process of pygophore relatively broad in lateral view (Figs 10B, 11B); lateral arm of proctiger moderately curve and slender in ventral view (Fig. 8B), with a weakly developed subapical process in lateral view (Fig. 10B).

#### Comparative notes.

*A.brevifasciata* Miyamoto, 1967 is most similar to *A.fumi* Esaki, 1925, see comparative notes in Polhemus and Andersen (1984).

#### Distribution.

China: Hainan (Zettel et al. 2007), Guangxi (Fig. 18). Thailand; Vietnam; Peninsular Malaysia; Singapore; Indonesia: Sumatra (Polhemus and Andersen 1984).

#### Remarks.

This species was previously recorded in Hainan (Zettel et al. 2007), but we have not found it in this

#### distribution.

##### ﻿New record for China

### ﻿ Amemboa burmensis

Taxon classificationAnimaliaHemipteraGerridae

J. Polhemus & Andersen, 1984

3BF0DC45-ADC1-5608-B7AB-52D116CCB190

Figs 2D , 3C , 4C, G , 6C, D , 7C , 8C , 9C , 10C , 11C , 12D , 13 , 14 , 15B , 18

#### Material examined.

5 ♂♂, 6 ♀♀ (apterous), China, Yunnan Province, De-hong Autonomous Prefecture, Na-bang Village; 24°42'5.8"N, 97°34'25.0"E; 207 m a.s.l.; 15 Apr. 2023; Mu Qiao, Ze-zhong Jin and Zi-he Li leg. (NKUM).

#### Diagnosis.

**Figure 13.**
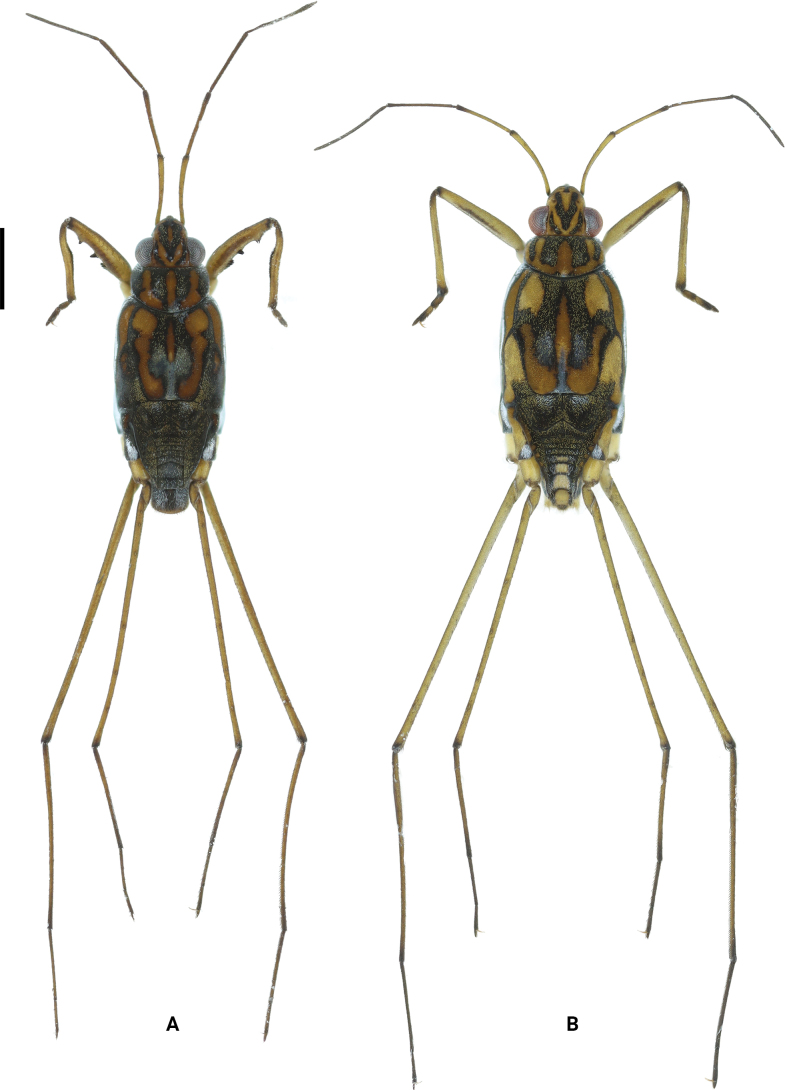
Habitus of *Amemboaburmensis* J. Polhemus & Andersen, 1984, apterous form in dorsal view **A** male **B** female. Scale bar: 1 mm.

**Figure 14.**
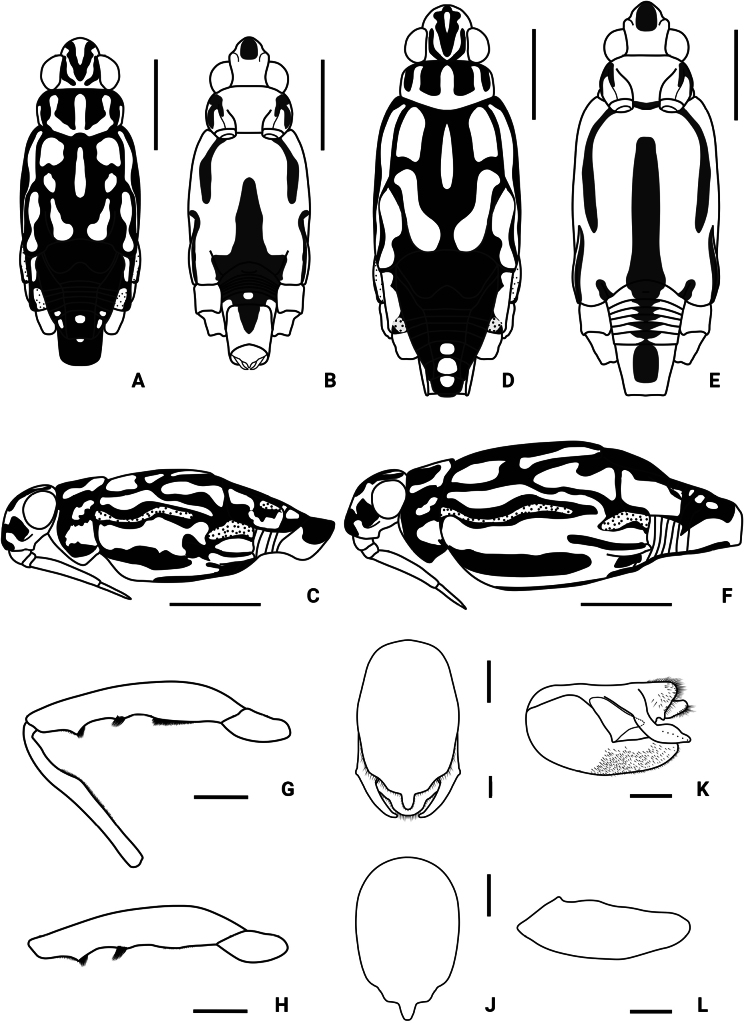
Morphological features of *Amemboaburmensis* J. Polhemus & Andersen, 1984 **A** body of male, dorsal view **B** body of male, ventral view **C** body of male, lateral view **D** body of female, dorsal view **E** body of female, ventral view **F** body of female, lateral view **G, H** left forelegs of different males, dorsal view **I** pygophore and proctiger of male, ventral view **J** pygophore of male, ventral view **K** pygophore and proctiger of male, lateral view **L** pygophore of male, lateral view. Scale bars: 1 mm (**A–F**); 0.2 mm (**G, H**); 0.1 mm (**I–L**).

Color pattern as shown in Figs 2D, 3C, 4C, G, 12D, 13, 14A–F. Males: profemur moderately incrassate; ventral side of the profemur with two tufts of dark setae on apical 1/2 and an additional elongate crest of dark setae on basal 1/2 (Figs 6C, 14G) (sometimes raised, as in Figs 6D, 14H); protibia slightly curved and with a tumescence on basal 1/3 (Figs 6C, 14G); abdominal segment VIII relatively long (Fig. 7C); pygophore posteriorly with a short knob-like median process, and with a pair of blunt processes on both sides of the median process in ventral view (Figs 8C, 9C, 14I, J); median process of pygophore relatively broad in lateral view (Figs 10C, 11C, 14K, L). Lateral arm of proctiger relatively straight in ventral view (Figs 8C, 14I), distinctly broadened subapically in lateral view (Figs 10C, 14K), forming a blunt process (Figs 10C, 14K).

#### Comparative notes.

*Amemboaburmensis* is most similar to *A.kumari* (Distant, 1910) and *A.cambodiana* D. Polhemus, 2017, see comparative notes in Polhemus and Andersen (1984) and Polhemus (2017).

#### Distribution.

China: Yunnan (Fig. 18). Myanmar: Shigbwiyang (Polhemus and Andersen 1984).

#### Habitat.

**Figure 15.**
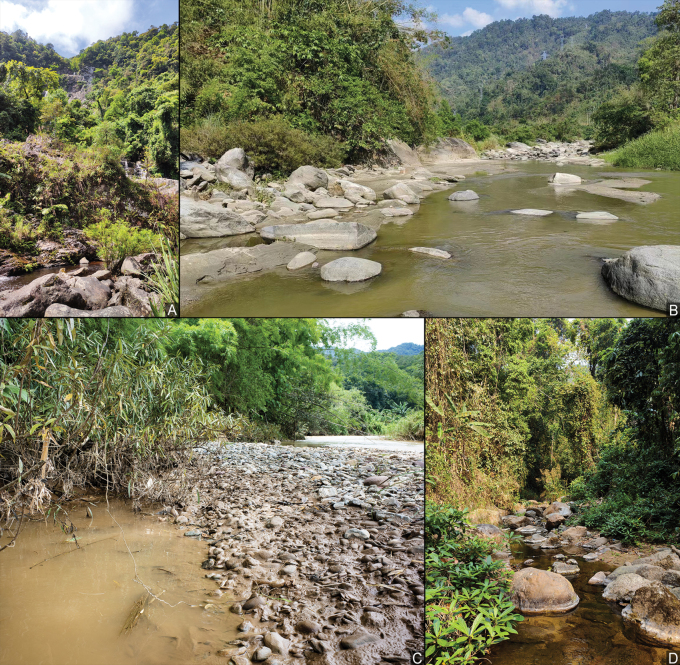
Habitat of *Amemboa* spp. **A** habitat of *A.hainanica* sp. nov., a tranquil pool at the base of the waterfall **B** habitat of *A.burmensis* J. Polhemus & Andersen, 1984, a wide, rocky river **C** habitat of *A.riparia* J. Polhemus & Andersen, 1984, a muddy pool beside the river **D** habitat of *A.riparia*, a small stream in the jungle.

We found *A.burmensis* inhabiting stagnant pools at the edges of a wide river (Fig. 15B), located near the lower altitudes of the forest in Tong-bi-guan, Yunnan.

#### Remarks.

In the original description of *A.burmensis*, Polhemus and Andersen (1984) described the profemur of males as follows: “Fore femur moderately incrassate in basal 1/2 (fig. 36), with an elongate patch of short dark hairs on ventral side”. However, among the specimens collected from China, this elongate tuft of setae is either more distinct than the original description (Fig. 6C) or nearly absent (Fig. 6D). We speculate that this may be caused by wear of the setae, or there may be two phenotypes of this species.

### ﻿ Amemboa cristata

Taxon classificationAnimaliaHemipteraGerridae

J. Polhemus & Andersen, 1984

8D9A29B1-2DEA-56C6-A578-AA495F8320FA

Figs 2E , 3D , 6E , 7D , 8D , 9D , 10D , 11D , 12E , 18

#### Material examined.

3 ♂♂, 4 ♀♀ (apterous), Thailand, Mae Hong Son Province, Pai, Thung Yao, Pam Bok Waterfall; 19°19'14.5"N, 98°24'18.6"E; 549 m a.s.l.; 26 Aug. 2018; Zhen Ye and Juan-juan Yuan leg. (NKUM).

#### Diagnosis.

Color pattern as shown in Figs 2E, 3D, 12E. Males: profemur moderately incrassate (Fig. 6E); ventral side of the profemur with one elongate crest of dark setae on apical 1/2 and an additional elongate crest of dark setae on basal 1/2 (Fig. 6E); protibia slightly curved and with a tumescence on basal 1/3 (Fig. 6E); abdominal segment VIII relatively long (Fig. 7D); pygophore posteriorly with a bifid median process and a pair of strongly produced lateral processes in ventral view (Figs 8D, 9D); median process of pygophore relatively narrow in lateral view (Figs 10D, 11D); lateral arm of proctiger relatively curved and slender in ventral view (Fig. 8D), without subapical process in lateral view (Fig. 10D)

#### Comparative notes.

*Amemboacristata* is most similar to *A.incurvata*; see comparative notes in Polhemus and Andersen (1984)

#### Distribution.

China: Yunnan (Zettel et al. 2007). Thailand; Vietnam; Peninsular Malaysia (Polhemus and Andersen 1984; Zettel and Chen 1996).

#### Remarks.

Zettel et al. (2007) first reported the distribution of *A.cristata* in Xi-shuang-ban-na, Yunnan. In this study, all the examined specimens and those used in the Figures are from Thailand.

### ﻿ Amemboa esakii

Taxon classificationAnimaliaHemipteraGerridae

J. Polhemus & Andersen, 1984

328F4BA5-86D5-553B-A17C-5356D9B2190E

Figs 2F , 3E , 6F , 7E , 8E , 9E , 10E , 11E , 12F , 18

#### Material examined.

2 ♂♂, 4 ♀♀ (apterous), China, Taiwan Island, Gao-xiong City, Liu-gui District; 23°0'17.1"N, 120°39'32.7"E; 262 m a.s.l.; 12 Sep. 2017; Juan-juan Yuan leg. (NKUM) • 14 ♂♂, 12 ♀♀ (apterous), China, Taiwan Island, Ping-dong County, Che-cheng Village, Si-chong-xi; 22°5'29.9"N, 120°45'44.9"E; 18 Nov. 2011; Wen-jun Bu leg. (NKUM) • 1 ♂, 4 ♀♀ (apterous), China, Taiwan Island, Ping-dong County, Man-zhou Village, Lan-ren-xi; 22°2'31.5"N, 120°51'35.6"E; 9 Nov. 2016; Hua-xi Liu leg. (NKUM) • 13 ♂♂, 5 ♀♀ (apterous), China, Taiwan Island, Ping-dong County, Mu-dan Village, Shou-ka-lin-dao; 22°14'55.3"N, 120°50'44.2"E; 15 Jun. 2013; Zhen Ye leg. (NKUM) • 7 ♂♂, 5 ♀♀ (apterous), China, Taiwan Island, Ping-dong County, Shi-zi Village, Li-long-shan; 22°10'02.3"N, 120°44'32.5"E; 18 Jun. 2013; Zhen Ye leg. (NKUM) • 3 ♂♂, 6 ♀♀ (apterous), China, Taiwan Island, Tai-dong County, Bei-nan Village; 22°46'51.9"N, 121°4'28.9"E; 14 Jun. 2013; Zhen Ye leg. (NKUM).

#### Diagnosis.

Color pattern as shown in Figs 2F, 3E, 12F. Males: profemur moderately incrassate; ventral side of the profemur with two tufts of dark setae on apical 1/2 (Fig. 6F); protibia slightly curved and with an indistinct tumescence on basal 1/3 (Fig. 6F); abdominal segment VIII relatively long (Fig. 7E); pygophore posteriorly with a blunt T-shaped median process and a pair of right-angled lateral processes in ventral view (Figs 8E, 9E); median process of pygophore relatively narrow in lateral view (Figs 10E, 11E); posterior margin of pygophore nearly truncate ventrally (Figs 8E, 9E); lateral arm of proctiger relatively curved in ventral view (Fig. 8E), without subapical process in lateral view (Fig. 10E).

#### Comparative notes.

*Amemboaesakii* is distinct from all congeners in having a T-shaped median process and a truncate posterior margin of pygophore in ventral view (Figs 8E, 9E).

#### Distribution.

China: Taiwan (Fig. 18).

### ﻿ Amemboa fumi

Taxon classificationAnimaliaHemipteraGerridae

Esaki, 1925

8EDD6F98-3167-581F-AC08-A925273F1235

Figs 2G , 3F , 4D, H , 6G , 7F , 8F , 9F , 10F , 11F , 12G , 18

#### Material examined.

3 ♂♂ (apterous), China, Taiwan Island, Nan-tou County, Ren-ai Village, Nan-shan-xi; 24°1'36.9"N, 121°5'21.0"E; 6 Jun. 2013; Zhen Ye leg. (NKUM) • 6 ♂♂, 5 ♀♀ (apterous), China, Taiwan Island, Xin-bei County, Wu-lai District, Xin-xian-bu-dao; 24°50'25.1"N, 121°32'15.0"E; 197 m a.s.l.; 6 Sep. 2017; Juan-juan Yuan leg. (NKUM) • 2 ♂♂ (apterous), China, Taiwan Island, Nan-tou County, Yu-chi Village; 23°50'58.9"N, 120°55'37.0"E; 6 Nov. 2016; Hua-xi Liu leg. (NKUM).

#### Diagnosis.

Color pattern as shown in Figs 2G, 3F, 4D, H, 12G. Males: profemur moderately incrassate (Fig. 6G); ventral side of the profemur with two tufts of dark setae on the apical 1/2 (Fig. 6G); protibia slightly curved and with an indistinct tumescence on basal 1/3 (Fig. 6G); abdominal segment VIII relatively short (Fig. 7F); pygophore posteriorly with a digitate median process in ventral view, also with a pair of blunt processes at lateral sides of the median process, without distinct angular lateral process (Figs 8F, 9F); median process of pygophore relatively narrow in lateral view (Figs 10F, 11F); lateral arm of proctiger relatively curved and slender in ventral view (Fig. 8F), without subapical process in lateral view (Fig. 10F).

#### Comparative notes.

See comparative notes under *A.hainanica* sp. nov.

#### Distribution.

China: Taiwan (Fig. 18).

### ﻿ Amemboa riparia

Taxon classificationAnimaliaHemipteraGerridae

J. Polhemus & Andersen, 1984

82A2508E-3766-56BF-90FC-B1C46CD61C23

Figs 2H, I , 3G, H , 6H, I , 7G, H , 8G, H , 9G, H , 10G, H , 11G, H , 12H, I , 15C, D , 17 , 18

#### Material examined.

1 ♂, 1 ♀ (apterous), China, Yunnan Province, Xi-shuang-ban-na Autonomous Prefecture, Jing-hong City, Pu-wen Town; 22°30'36.5"N, 101°3'55.5"E; 880 m a.s.l.; 25 Apr. 2011; Zhen Ye leg. (NKUM) • 7 ♂♂, 6 ♀♀ (apterous), China, Yunnan Province, Xi-shuang-ban-na Autonomous Prefecture, Jing-hong City, Man-dian Village, Na-ban-he Nature Reserve; 22°7'48.5"N, 100°39'46.1"E; 629 m a.s.l.; 28 Jul. 2016; Zhen Ye leg. (NKUM) • 1 ♂ (apterous), China, Yunnan Province, Xi-shuang-ban-na Autonomous Prefecture, Jing-hong City, Meng-la County, Meng-lun Town, Ba-ka-xiao-zhai Village; 21°57'57.0"N, 101°12'16.2"E; 747 m a.s.l.; 5 Jul. 2018; Juan-juan Yuan and Yan-fei Li leg. (NKUM) • 1 ♂ (apterous), China, Yunnan Province, Xi-shuang-ban-na Autonomous Prefecture, Jing-hong City, Meng-la County, Mo-han Town; 21°11'51.0"N, 101°41'55.8"E; 17 Aug. 2014; Zhen Ye leg. (NKUM) • 3 ♀♀ (apterous), China, Yunnan Province, Xi-shuang-ban-na Autonomous Prefecture, Jing-hong City, Meng-la County, Yao-qu-yao-zu Township; 21°42'57.0"N, 101°32'32.2"E; 758 m a.s.l.; 26 Jul. 2016; Zhen Ye leg. (NKUM) • 4 ♂♂, 3 ♀♀ (apterous), China, Yunnan Province, Xi-shuang-ban-na Autonomous Prefecture, Jing-hong City, Man-dian Village, Man-dian-pu-bu; 22°7'45.1"N, 100°40'01.9"E; 660 m a.s.l.; 20 Apr. 2023; Ze-zhong Jin and Zi-he Li leg. (NKUM) • 3 ♂♂, 2 ♀♀ (apterous), China, Yunnan Province, Pu-er City, Meng-lian County, Meng-ma Town, Mang-yun Village; 22°13'50.5"N, 99°21'30.1"E; 887 m a.s.l.; 20 Jul. 2016; Zhen Ye leg. (NKUM) • 2 ♂♂, 1 ♀ (apterous), China, Yunnan Province, Xi-shuang-ban-na Autonomous Prefecture, Jing-hong City, Pu-wen Town, Cai-yang-he; 22°2'43.2"N, 100°56'10.3"E; 25 Apr. 2011; Rui Wang leg. (NKUM) • 1 ♂ (apterous), China, Yunnan Province, Pu-er City, Si-mao District, Nan-dao-he; 22°36'45.9"N, 100°59'46.6"E; 990 m a.s.l.; 15 Jul. 2018; Juan-juan Yuan leg. (NKUM) • 1 ♂ (apterous), 1 ♀ (macropterous), China, Yunnan Province, Xi-shuang-ban-na Autonomous Prefecture, Jing-hong City, Nan-la County; 21°44'25.6"N, 101°18'8.0"E; 28 Apr. 2011; Rui Wang leg. (NKUM).

#### Diagnosis.

Color pattern as shown in Figs 2H, I, 3G, H, 12H, I. Males: profemur incrassate (Fig. 6H, I); ventral side of the profemur with two indistinct tufts of short setae on the apical 1/2 and an additional large, elongate crest of dark setae on basal 1/2 (Fig. 6I), occasionally also with a tuft of short setae in the middle (Fig. 6H); protibia slightly curved and with a distinct tumescence on basal 1/2 (Fig. 6H, I); abdominal segment VIII relatively long (Fig. 7G, H); in ventral view, pygophore posteriorly with a short knob-like median process, a pair of distinct angular lateral processes, and a pair of indistinct blunt processes between the median process and lateral processes (Figs 8G, H, 9G; H); median process of pygophore relatively broad in lateral view (Figs 10G, H, 11G, H); lateral arm of proctiger with an angular process laterally and basally curved in ventral view (Fig. 8G, H), and in lateral view with a distinctly pointed subapical process (Fig. 10G, H).

#### Comparative notes.

*A.riparia* is most similar to *A.lyra* (Paiva, 1918), see comparative notes in Polhemus and Tran (2012).

#### Distribution.

China: Yunnan (Fig. 18). Thailand; Laos; Vietnam; Peninsular Malaysia; Singapore (Polhemus and Tran 2012).

#### Habitats.

We observed *A.riparia* inhabiting the edges of streams with extremely slow water currents (Fig. 15D), as well as on completely still ponds (Figs 15C, 17A–C).

**Figure 16.**
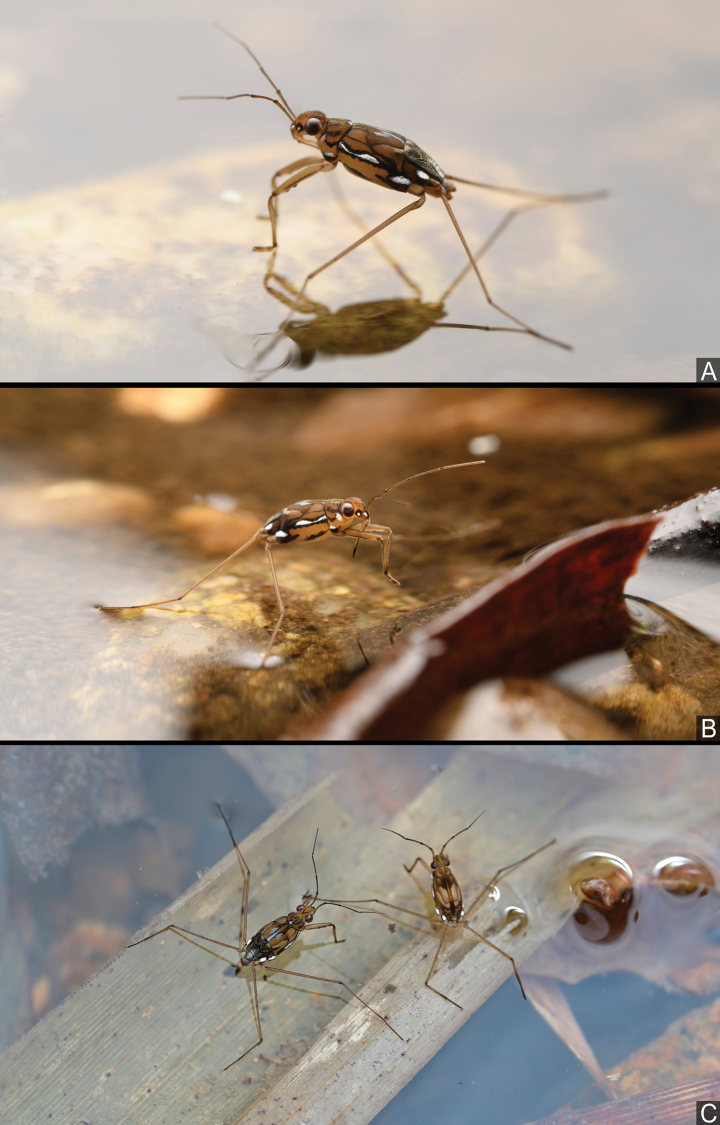
*Amemboahainanica* sp. nov., live habitus in situ **A** an apterous male moving on the water surface **B** an apterous male cleaning its forelegs **C** an apterous male and a nymph standing on the water surface (photographed by Fan Gao). Images not to scale.

**Figure 17.**
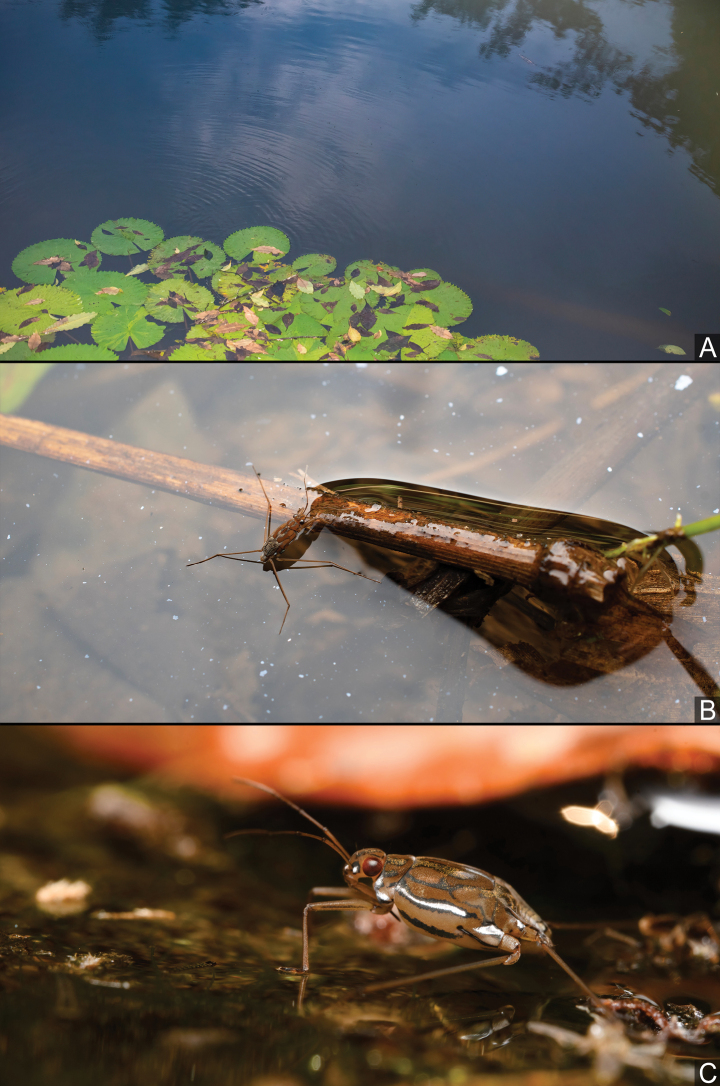
*Amemboariparia* J. Polhemus & Andersen, 1984, habitat and live habitus in situ **A** habitat of *A.riparia*, at the banks of a still pond next to the ravine rainforest **B** apterous male, dorsal view **C** apterous female, lateral view. Images not to scale.

**Figure 18.**
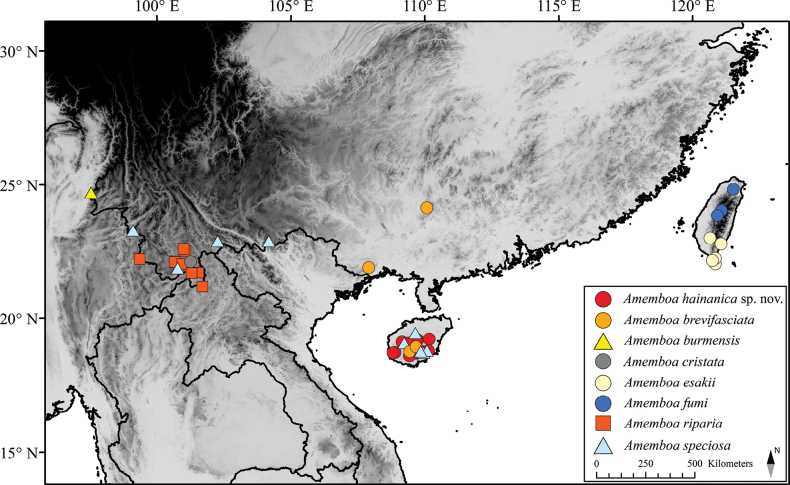
Distribution map of *Amemboa* spp. of China.

#### Remarks.

Based on the reasons listed in the introduction, we believe that the “*A.lyra* (Paiva, 1918)” reported by Cheng et al. (2006) is actually *A.riparia*. Therefore, we conclude that *A.lyra* has not been recorded in China.

The differences between *A.riparia* and *A.lyra* have been extensively discussed by Polhemus and Tran (2012). Moreover, Polhemus and Tran (2012) discovered specimens of *A.riparia* with a small tuft of setae in the middle of the profemora. These specimens were collected from northern Thailand and northern Vietnam, exhibiting genitalic segments correspond to *A.riparia* (Polhemus and Tran 2012). Additionally, the middle setae tufts of profemur also slightly differed from those of *A.lyra* (Polhemus and Tran 2012).

We found several specimens with similar morphology characteristics in southern Yunnan (Figs 2H, 3G, 6G, 7G, 8G, 9G, 10G, 11G, 12H), including a couple from Jinghong City and a male from Pu’er City. These specimens also have a small tuft of setae in the middle of the male profemora. The lateral process of the pygophore (Figs 8G, 9G) and the processes on the lateral arms of the proctiger (Fig. 10g) both aligned more closely with those of *A.riparia* (see Polhemus and Andersen 1984: figs 77, 78; also see Polhemus and Tran 2012: fig. 4) rather than *A.lyra* (see Polhemus and Andersen 1984: figs 74, 75; also see Polhemus and Tran 2012: fig. 2). Given that we only found a few individuals and not a large population, we are more inclined to believe that these specimens belong to *A.riparia*, with the male profemur differences resulting from occasional mutations.

### ﻿ Amemboa speciosa

Taxon classificationAnimaliaHemipteraGerridae

J. Polhemus & Andersen, 1984

A30A85C0-A76F-572B-B026-B0066EC623F8

Figs 2J , 3I , 6J , 7I , 8I , 9I , 10I , 11I , 12J , 18

#### Material examined.

5 ♂♂, 5 ♀♀ (apterous), China, Hainan Province, Dan-zhou City, Lan-yang Town, Lian-hua-ling; 19°27'6.8"N, 109°38'48.5"E; 213 m a.s.l.; 21 Jul. 2017; Zhen Ye leg. (NKUM) • 5 ♂♂, 5 ♀♀ (apterous), China, Hainan Province, Ling-shui County, Da-xing Waterfall; 18°43'54.9"N, 109°57'1.0"E; 170 m a.s.l.; 8 Aug. 2017; Zhen Ye leg. (NKUM) • 4 ♂♂ (apterous), China, Hainan Province, Qiong-zhong County, Chang-xing Village; 18°48'04.2"N, 110°4'38.2"E; 158 m a.s.l.; 11 Aug. 2017; Zhen Ye leg. (NKUM) • 1 ♂, 2 ♀♀ (apterous), China, Hainan Province, Chang-jiang County, Ba-wang-ling Nature Reserve; 19°5'0.2"N, 109°13'34.0"E; 457 m a.s.l.; 25 Jul. 2017; Zhen Ye leg. (NKUM) • 1 ♂ (apterous), China, Hainan Province, Qiong-zhong County, Beng-ling Village; 18°46'42.7"N, 109°50'29.3"E; 253 m a.s.l.; 8 Aug. 2017; Zhen Ye leg. (NKUM) • 2 ♂♂, 2 ♀♀ (apterous), China, Yunnan Province, Hong-he Autonomous Prefecture, Lv-chun County, Huang-lian-shan; 22°53'7.0"N, 102°16'16.2"E; 1500–1800 m a.s.l.; 22 Apr. 2011; Rui Wang leg. (NKUM) • 6 ♂♂, 7 ♀♀ (apterous), China, Yunnan Province, Wen-shan Autonomous Prefecture, Ma-guan County, Bao-bao-zhai Village; 22°53'7.8"N, 104°10'59.9"E; 813 m a.s.l.; 8 Aug. 2020; Zhen Ye leg. (NKUM) • 4 ♂♂, 5 ♀♀ (apterous), China, Yunnan Province, Lin-cang City, Cang-yuan County, Ban-hong Town, Nan-gun-he; 23°17'46.7"N, 99°6'18.6"E; 7 May 2011; Zhen Ye leg. (NKUM) • 8 ♂♂, 3 ♀♀ (apterous), China, Yunnan Province, Xi-shuang-ban-na Autonomous Prefecture, Jing-hong City, Man-he-hui Waterfall; 21°53'49.5"N, 100°46'3.0"E; 603 m a.s.l.; 23 Nov. 2018; Zhen Ye leg. (NKUM).

#### Diagnosis.

Color pattern as shown in Figs 2J, 3I, 12J. Males: profemur incrassate (Fig. 6J); ventral side of the profemur with two tufts of dark setae on the apical 1/2 and an additional large, elongate crest of dark setae on basal 1/2 (Fig. 6J); protibia slightly curved and with an indistinct tumescence medially (Fig. 6J); abdominal segment VIII relatively long (Fig. 7I); pygophore posteriorly with a median broad triangular process in ventral view, without other special modifications (Figs 8I, 9I); median process of pygophore relatively broad in lateral view (Figs 10I, 11I). Lateral arm of proctiger relatively simple, tapering towards narrow apex in ventral and lateral views (Figs 8I, 10I), relatively broadened proximally and without subapical process in lateral view (Fig. 10I).

#### Comparative notes.

*Amemboaspeciosa* J. Polhemus & Andersen, 1984 is most similar to *A.intermedia* Zettel & Chen, 1996; see comparative notes in Zettel and Chen (1996).

#### Distribution.

China: Hainan, Yunnan (Fig. 18). Thailand (Zettel and Chen 1997); Laos (Zettel 1998); Vietnam (Polhemus and Andersen 1984).

### ﻿Key to species of *Amemboa* Esaki, 1925 of China (Males)

**Table:** 

1	Profemur with 2 elongate crests of dark setae (Fig. 6E). Median process of pygophore posteriorly bifid (Fig. 9D)	***A.cristata* J. Polhemus & Andersen, 1984**
–	Profemur with < 2 elongate crests of dark setae (Fig. 6A–D, F–J). Median process of pygophore posteriorly not bifid (Fig. 9A–C, E–I)	**2**
2	Ventral surface of profemur with an elongate patch of dark setae on basal 1/3 (Fig. 6C, H–J)	**3**
–	Ventral surface of profemur without dark setae on basal part (Fig. 6A, B, F, G)	**5**
3	Protibia with a relatively distinct process (Fig. 6H, I). Lateral process of pygophore angular and distinctly produced in ventral view (Fig. 9G, H). Lateral arm of proctiger with a distinct lateral process basally in ventral view (Fig. 8G, H), also with a distinctly pointed process in lateral view (Fig. 10G, H)	***A.riparia* J. Polhemus & Andersen, 1984**
–	Protibia with a relatively indistinct process (Fig. 6C, D, J). Lateral process of pygophore blunt, weakly produced in ventral view (Fig. 9C, I). Lateral arm of proctiger without a distinct lateral process basally in ventral view (Fig. 8C, I), also without a distinctly pointed process in lateral view (Fig. 10C, I)	**4**
4	Median process of pygophore knob-like in ventral view (Fig. 9C). Lateral arm of proctiger relatively wide in lateral view (Fig. 10C)	***A.burmensis* J. Polhemus & Andersen, 1984**
–	Median process of pygophore broadly triangular in ventral view (Fig. 9I). Lateral arm of proctiger relatively narrow in lateral view (Fig. 10I)	***A.speciosa* J. Polhemus & Andersen, 1984**
5	Protibia with a distinct angular process on basal part (Fig. 6F). Abdominal segment VIII relatively long (Fig. 7E). Pygophore posteriorly with a blunt T-shaped median process in ventral view (Fig. 9E). Posterior margin of pygophore truncated, not tapering towards the end in ventral view (Fig. 9E)	***A.esakii* J. Polhemus & Andersen, 1984**
–	Protibia with a weakly developed blunt process on basal part (Fig. 6A, B, G). Abdominal segment VIII relatively short (Fig. 7A, B, F). Pygophore posteriorly with a digitate process in ventral view (Fig. 9A, B, F). Posterior margin of pygophore tapering towards the digitate median process in ventral view (Fig. 9A, B, F)	**6**
6	Pygophore lacks any processes other than median process in ventral view (Fig. 9B). Posterior margin of pygophore relatively broad and blunt in ventral view (Fig. 9B)	***A.brevifasciata* Miyamoto, 1967**
–	Pygophore with a pair of small and blunt lateral process in ventral view (Fig. 9A, F). Posterior margin of pygophore relatively narrow and digitate in ventral view (Fig. 9A, F)	**7**
7	Lateral black stripes on mesonotum relatively broad in dorsal view (Figs 2G, 12G). Lateral arm of proctiger without a distinct subapical process in lateral view (Fig. 10F). Endemic to Taiwan Island	***A.fumi* Esaki, 1925**
–	Lateral black stripes on mesonotum relatively slim (Figs 2A, 12A) or nearly lost (Figs 2B, 12B) in dorsal view. Lateral arm of proctiger with a distinct subapical process in lateral view (Fig. 10A). Endemic to Hainan Island	***A.hainanica* sp. nov.**

## Supplementary Material

XML Treatment for Amemboa hainanica

XML Treatment for Amemboa brevifasciata

XML Treatment for Amemboa burmensis

XML Treatment for Amemboa cristata

XML Treatment for Amemboa esakii

XML Treatment for Amemboa fumi

XML Treatment for Amemboa riparia

XML Treatment for Amemboa speciosa

## ﻿Additional information

### Author ORCIDs

Zhaoqi Leng https://orcid.org/0009-0005-8391-6077

Beichen Zhang https://orcid.org/0009-0004-1618-6134

Zezhong Jin https://orcid.org/0000-0001-7223-770X

Zhen Ye https://orcid.org/0000-0003-2327-1869

### Data availability

All of the data that support the findings of this study are available in the main text.
